# Long non‐coding RNA TNRC6C‐AS1 promotes methylation of STK4 to inhibit thyroid carcinoma cell apoptosis and autophagy via Hippo signalling pathway

**DOI:** 10.1111/jcmm.14728

**Published:** 2019-10-27

**Authors:** Xinzhi Peng, Chengcheng Ji, Langping Tan, Shaojian Lin, Yue Zhu, Miaoyun Long, Dingyuan Luo, Honghao Li

**Affiliations:** ^1^ Department of Thyroid Surgery The Sun Yat‐Sen Memorial Hospital Sun Yat‐Sen University Guangzhou China; ^2^ Department of Cardiology The First Affiliated Hospital of Sun Yat‐Sen University Guangzhou China

**Keywords:** Hippo signalling pathway, long non‐coding RNA TNRC6C‐AS1, methylation, STK4, thyroid carcinoma

## Abstract

The role of long non‐coding RNAs (lncRNAs) in thyroid carcinoma (TC), the most frequent endocrine malignancy, has been extensively examined. This study investigated effect of interaction among lncRNA TNRC6C‐AS1, serine/threonine‐protein kinase 4 (STK4) and Hippo signalling pathway on TC. Initially, lncRNA TNRC6C‐AS1 expression in TC tissues was detected. To explore roles of lncRNA TNRC6C‐AS1, STK4 and Hippo signalling pathway in TC progression, their expressions were altered. Interaction between lncRNA TNRC6C‐AS1 and STK4, STK4 promoter methylation, or Hippo signalling pathway was verified. After that, a series of experiments were employed to evaluate in vitro ability of apoptosis, proliferation and autophagy of TC cells and in vivo tumorigenicity, and tumour growth of TC cells. lncRNA TNRC6C‐AS1 was highly expressed while STK4 was poorly expressed in TC tissues. LncRNA TNRC6C‐AS1 promoted the STK4 methylation and down‐regulated STK4 expression, which further activated the Hippo signalling pathway. STK4 silencing was observed to promote the proliferation ability of TC cells, inhibit the apoptosis and autophagy abilities, as well as enhance the tumorigenicity and tumour growth. Moreover, the in vitro proliferation ability as well as the in vivo tumorigenicity and tumour growth of TC cells were inhibited after the blockade of Hippo signalling pathway, while the apoptosis and autophagy abilities were promoted. The results demonstrate that the lncRNA TNRC6C‐AS1 increases STK4 promoter methylation to down‐regulate STK4 expression, thereby promoting the development of TC through activation of Hippo signalling pathway. It highlights that lncRNA TNRC6C‐AS1 may be a novel therapeutic target for the treatment of TC.

## 1 INTRODUCTION

Thyroid carcinoma (TC) is considered as one of the most common malignancy of endocrine system.^1^ In the last decade, the incidence of TC has dropped by about 2% annually in men, while the mortality rate dropped by about 1.5% annually in both men and women.^2^ TC can be subdivided into several types, including differentiated (papillary, follicular, Hurthle cell), which accounts for about 90% of TC cases, medullary TC and anaplastic TC.^3^ Radiation to the thyroid gland during our childhood, age and family disease history are the several main risk factors to high‐level differentiated TC.^4^ Despite the progress for the treatment of TC, the 5‐year cancer‐specific survival rate remains low.^5^ Therefore, it is important to identify the specific biomarkers that can be used in diagnosis of prognosis in TC patients.

Long non‐coding RNA (lncRNA) dysfunction is related to a wide range of diseases, such as cancer, neurodegeneration, cardiovascular disease and neurasthenia.^6^ For example, overexpressed lncRNA NR_036575.1 would lead to the proliferation and migration of papillary TC.^7^ In order to discover the genes that might be correlated with TC, the Gene Expression Omnibus (GEO) database was employed to retrieve the lncRNA trinucleotide repeat containing 6C (TNRC6C)‐AS1 was overexpressed in TC cells. Therefore, lncRNA TNRC6C‐AS1 was the main focus of our study. LncRNA TNRC6C‐AS1 has been reported to play a tumorigenic role in the tumour occurrence and invasive ability of papillary thyroid carcinoma (PTC).^8^ A new study has found that lncRNA TNRC6C‐AS1 affects the development of TC by regulating UNC5B expression as a competitive endogenous RNA of microRNA‐129‐5p (miR‐129‐5p).^9^ The complementary pairing sites in the promoter regions of lncRNA TNRC6C‐AS1 and serine/threonine‐protein kinase 4 (STK4) genes were analysed by bioinformatics. STK4, a multifunctional protein, is an essential kinase of the Hippo signalling pathway for tumour inhibition in the treatment of cancers with poor prognosis.^10^ STK4 is considered as a tumour suppressor in hepatocellular carcinoma, breast cancer and lymphoma that regulates cell apoptosis.^11^, ^12^, ^13^ Hippo signalling pathway was initially discovered by genetic studies in *Drosophila* as a regulator of organ size, limiting cell number via the regulation of cell proliferation and apoptosis.^14^ Hippo signalling pathway is responsible for controlling organ size by regulating cell proliferation, apoptosis, stem‐cell self‐renewal and tumorigenesis of TC cells.^15^ From all that mentioned above, we hypothesize that lncRNA TNRC6C‐AS1 in TC can affect the proliferation, apoptosis and autophagy in TC through STK4 regulation and the Hippo signalling pathway. Thus, this study was aimed to determine the roles of lncRNA TNRC6C‐AS1, STK4 and Hippo signalling pathway in apoptosis and autophagy of TC cells.

## 2 MATERIALS AND METHODS

### 2.1 Ethical statement

All patients in this study signed an informed consent, which was in accordance with the Helsinki Declaration and approved by the ethics committee of the Sun Yat‐Sen Memorial Hospital. This experiment animal‐related programme has been approved by the experimental animal ethics Committee and conforms to the relevant regulations of the national experimental animal welfare ethics. Significant efforts were made in order to minimize the number of animals used as well as their respective suffering. The study was conducted with the approval of the Animal Ethics Committee of the Sun Yat‐Sen Memorial Hospital, Sun Yat‐Sen University.

### 2.2 Microarray analysis

The GEO database was used to retrieve the TC‐related microarray data and annotation probe file, obtained by examination of Affymetrix Human Genome U133 Plus 2.0 Array. Each microarray data set was processed using the Affy package of R software.^16^ Then, the linear model‐Empirical Bayes statistical method in the Limma package was combined with the traditional *t* test to perform non‐specific filtering of the microarray data and to screen the differentially expressed miRs and lncRNAs.^17^ The Multi Expert Matrix (MEM, http://biit.cs.ut.ee/mem/) website was employed to predict the differentially expressed lncRNAs. The Kyoto Encyclopedia of Genes and Genomes (KEGG) enrichment analysis was conducted using WebGesitat database (http://www.webgestalt.org) to determine the most important biochemical metabolic pathway and signalling pathway the gene involved.^18^ Blast comparison results showed that the promoter regions of lncRNA TNRC6C‐AS1 and STK4 had base complementary pairing binding sites.

### 2.3 Tissue samples and cell lines

A total of 54 cases of TC tissues were obtained from specimens resected after TC surgery in the Sun Yat‐Sen Memorial Hospital, Sun Yat‐Sen University. The materials were taken and stored in the −80°C refrigerator immediately after the operation. Paracancerous tissues were taken from normal thyroid tissue, 1.0 cm away from the edge of TC tissues.

TC cell lines BCPAP (number BNCC338685), TPC‐1 (number BNCC337912), K1 (number BNCC337627), SW579 (number BNCC100182), FTC‐133 (number BNCC33799) and normal thyroid cell line Nthy‐ori 3‐1 (number BNCC340487) were all purchased from Beijing Beina Chuanglian Biotechnology Research Institute. The cells were cultured in Roswell Park Memorial Institute‐1640 medium (Gibco) containing 10% foetal bovine serum (FBS) (Gibco), placed and subcultured in a 5% CO_2_ incubator (Thermo) at 37°C. When the cell density reached 90%, cells were detached with 0.25% trypsin and subcultured at a ratio of 1:3.

### 2.4 Cell transfection and grouping

The cells were assigned into 8 groups: the blank group (without transfection with any plasmid), the negative control (NC) group (transfected with empty plasmid), the oelncRNA TNRC6C‐AS1 group (transfected with overexpressed lncRNA TNRC6C‐AS1 plasmid), the shlncRNA TNRC6C‐AS1 group (transfected with silenced lncRNA TNRC6C‐AS1 plasmid), the shSTK4 group (transfected with silenced STK4 plasmid), the shlncRNA TNRC6C‐AS1+shSTK4 group (cotransfected with silenced lncRNA TNRC6C‐AS1 plasmid and silenced STK4 plasmid), the RA‐V(1) group (Hippo signalling pathway inhibitor, purchased from Chinese Academy of Sciences, 100 μM), the shSTK4+RA‐V(1) group (cotransfected with RA‐V(1) and silenced STK4 plasmid). All plasmids were purchased from Shanghai GenePharma Co., Ltd..

### 2.5 Fluorescence in situ hybridization (FISH)

The subcellular localization of lncRNA TNRC6C‐AS1 in SW579 cells was identified by FISH. The following steps were performed using Ribo™ lncRNA FISH Probe Mix (Red) (Ruibo Biotech Co., Ltd.) according to the instructions from manufacturer's protocol. The SW579 cells were inoculated into a 6‐well culture plate covered with a coverslip for 1 d to reach about 80% confluency. Next, the cells were fixed in 1 mL 4% paraformaldehyde at room temperature. The cells were first treated with protease K (2 μg/mL), glycine and ethanolamine, then added with 250 μL pre‐hybridization solution and then incubated at 4°C for 1 hour. Subsequently, the cells were added with 250 μL hybridization solution containing probe (300 ng/mL) and hybridized at 42°C overnight. After the cells were washed with Phosphate‐Buffered Saline/Tween (PBST) 3 times, cells were stained using 4′,6‐Diamidino‐2‐Phenylindole (DAPI) (1:800) diluted with PBST, and then added to a 24‐well culture plate for 5 minutes. The cells were sealed with anti‐fluorescence quencher, and five different fields of view were selected to observe and photograph under the fluorescence microscope (Olympus).

### 2.6 Chromatin immunoprecipitation (ChIP)

ChIP kit (Millipore) was used to identify the enrichment of DNA methyltransferase1 (DNMT1), DNMT3a and DNMT3b in the promoter region of STK4. When the thyroid cells reached 70%‐80% fusion rate, 1% formaldehyde were added to fix the cells at room temperature for 10 minutes to immobilize and cross‐link the DNA and protein in the cells. After cross‐linking, the cells were randomly broken by ultrasonic treatment, 10 seconds each time with an interval of 10 seconds for 15 cycles to break cells into fragments of appropriate size. After that, the cells were centrifuged at 30 237 g at 4°C, the supernatant was then collected and aliquoted into three tubes. The positive control antibody RNA polymerase II, Immunoglobulin G (IgG) of normal mice with NC antibody and specific antibody DNMT1 of target protein (Abcam Inc, ab13537, Rabbit anti), DNMT3a (Abcam Inc, ab2850, Rabbit anti), DNMT3b (Abcam Inc, ab2851, Rabbit anti) were separately added to the cells and incubated overnight at 4°C. The protein agarose/sepharose was utilized to precipitate the endogenous DNA‐protein complexes. After centrifugation, the supernatant was aspirated and the non‐specific complex was washed, de‐cross‐linked at 65°C overnight, and then, DNA fragments were purified by phenol/chloroform extraction. The binding of DNMT1, DNMT3a and DNMT3b with STK4 promoter region was examined by STK4 promoter region specific primers (Table 1).

**Table 1:** Primer sequences

Gene	Primer sequence (5′‐3′)
STK4‐Methylation	F: GGATTTAGGATTTAGGTAGAGGTGC
	R: CCTAATCACGTAATTCGAACGTA
STK4‐Non‐Methylation	F: TTTAGGATTTAGGTAGAGGTGTGT
	R: CCCTAATCACATAATTCAAACATA

Abbreviations: F, forward primer; R, reverse primer; STK4, serine/threonine‐protein kinase4.

### 2.7 RNA‐binding protein co‐immunoprecipitation (RIP)

According to the instructions of Magna RIP RNA‐Binding Protein Immuno Preparation Kit (Millipore), the specific steps were as follows. Cells from the blank group, the oelncRNA TNRC6C‐AS1 group and the shlncRNA TNRC6C‐AS1 group were collected by cell scraper, washed twice with pre‐cooled PBS and added with 100 μL prepared lysis buffer containing protease inhibitor and ribonuclease inhibitor. The cells were centrifuged at 25 764 *g* for 3 minutes at 4°C after lysed on ice for 30 minutes. A small amount of supernatant was used as Input positive control and added with 1 μg corresponding antibodies: DNMT1 (ab13537), DNMT3a (ab2850) and DNMT3b (ab2851); about 10‐50 μL protein A/G‐beads were incubated overnight at 4°C and centrifuged at 1610 *g* at 4°C for 5 minutes, the supernatant was then discarded. The protein A/G‐beads precipitate was washed 3‐4 times with 1 mL lysis buffer, centrifuged at 179 *g* for 1 minutes at 4°C after each wash. A total of 15 μL 2 sodium dodecyl sulphate (2SDS) loading buffer was added and heated in boiling water for 10 minutes. RNA extraction method was used to separate and purify the related RNA from the precipitate. Reverse transcription quantitative polymerase chain reaction (RT‐qPCR) was performed with lncRNA TNRC6C‐AS1 as the specific primer to verify the interaction between DNMT1, DNMT3A, DNMT3B and lncRNA TNRC6C‐AS1. The antibodies used in RIP were as follows: rabbit anti‐human DNMT1 (1:100, ab13537), rabbit anti‐human DNMT3A (1:100, ab2850) and rabbit anti‐human DNMT3B (1:100, ab2851) and were mixed at room temperature for 30 minutes. Rabbit anti‐human IgG (1:100, ab109489) was used as NC. The antibodies were purchased from Abcam Inc.

### 2.8 Methylation‐specific PCR (MSP)

The DNA of cells was subjected to hydrosulfite treatment, whereas Wizard purified resin (Promega Corporation) was used to purify DNA samples. DNA was precipitated with ethanol and resuspended in water after NaOH treatment. PCR primers were designed according to Herman method. The sequences are shown in Table 1. STK4 methylation‐specific primers (M) and STK4 non–methylation specific primers (U) were purchased from Invitrogen (Invitrogen). The MSP amplification system procedure was as follows: pre‐denaturation at 94°C for 5 minutes, and 44 cycles of denaturation at 94°C for 15 seconds, at 60°C for 10 seconds and at 72°C for 9 seconds. Non–methylation specific PCR (UNMSP) amplification system procedure was as follows: pre‐denaturation at 94°C for 5 minutes, and 36 cycles of denaturation at 94°C for 15 seconds, at 60°C for 10 seconds and at 72°C for 8 seconds. The PCR products were electrophoresed by 1.5% agarose gel (Shanghai Sangon Biotechnology Co. Ltd.), stained with GelRed (nucleic acid dye, Biotium Co. Ltd.) and observed under the ultraviolet photograph gel apparatus (Bio‐Rad, Inc).

### 2.9 Immunofluorescence staining

After conventional treatment of transfected cells, the cells were counted and cultured in an immunofluorescence chamber with cell density of 2 × 10^5^ cells per well. When cell fusion rate reached about 90%, the cells were washed 3 times with PBS on ice. The cells were then fixed by 1 mL 4% paraformaldehyde into each well, standing at room temperature for 15 minutes. After cells were washed with PBS 3 times, the cells were perforated with 0.3% Triton. After 10 minutes, the cells were washed with PBS 3 times and sealed by goat serum, standing for 30 minutes. Subsequently, the cells were added with primary antibody prepared using PBS and placed at 4°C overnight. After 3‐time wash with PBS, the cells were added with secondary antibody and incubated for 1 hour at room temperature in the dark. The cells were then washed with PBS 3 times again and stained for 15 minutes by DAPI in the dark. The cells were then sealed by adding fluorescent quencher after 3‐time wash by PBS in the dark. Finally, the cells were observed and photographed under a fluorescence microscope.

### 2.10 RNA isolation and quantitation

Total RNA was extracted from tissues and cells by using TRIzol (Invitrogen). The nanodrop2000 micro ultraviolet spectrophotometer (1011U, nanodrop Technologies Inc) was used to detect the A260/A230 value, which was then used to determine the total RNA concentration and purity. Then, RNA was reversely transcribed into cDNA using the TaqMan MicroRNA Assays Reverse Transcription primer (4427975, Applied Biosystems, Inc). Primers for lncRNA TNRC6C‐AS1, STK4, large tumour suppressor kinase1 (LATS1) and Beclin1 were designed and synthesized using TaKaRa Biotechnology Ltd., as shown in Table 2. ABI7500 quantitative PCR instrument (7500, ABI Company) was employed to perform RT‐qPCR. The relative level of lncRNA TNRC6C‐AS1 used U6 as the internal reference. The relative expression of STK4, LATS1 and Beclin1 used glyceraldehyde‐3‐phosphate dehydrogenase (GAPDH) as an internal reference. LATS1 was the protein of involved in Hippo signalling pathway. The 2^−ΔΔCt^ represents the target gene expression ratio of the experimental group, the blank group and the NC group, and the formula was as follows: ΔΔCt = ΔCt (experimental group) − ΔCt (control and NC group), ΔCt = Ct (target gene) − Ct (internal reference). Ct was the cycle numbers when the real‐time fluorescence intensity reached the set threshold.

**Table 2:** Primer sequences for reverse transcription quantitative polymerase chain reaction

Gene	Primer sequence (5′‐3′)
LncRNA TNRC6C‐AS1	F: GGGTCTAGCCCACCCAATC
	R: ATGGGCTCAACAGGTCACAA
STK4	F: TGAAACTGAAACGCCAGGA
	R: TGCCAGAATCCATTTCATCC
LATS1	F: CCACCCTACCCAAAACATCTG
	R: CGCTGCTGATGAGATTTGAGTAC
Beclin1	F: TAGGATCCATGGAAGGGTCTAAGAC
	R: GCGAAGCTTTCATTTGTTATAAAAT
U6	F: CTCGCTTCGGCAGCACA
	R: AACGCTTCACGAATTTGCGT
GAPDH	F: ACCACCATGGAGAAGGCTGG
	R: CTCAGTAGCCCAGGATGC

Abbreviations: F, forward primer; GAPDH, Glyceraldehyde‐3‐phosphate dehydrogenase; LATS1, large tumour suppressor kinase1; LncRNA TNRC6C‐AS1, long non‐coding RNA TNRC6C‐AS1; MST1, mammalian sterile 20‐like kinase 1; R, reverse primer; STK4, serine/threonine‐protein kinase 4; YAP, yes‐associated protein.

### 2.11 Western blot analysis

Radio Immunoprecipitation Assay (BB‐3209, Bestbio Biotechnology Co. Ltd) was used to extract the total protein of the TC cells. The total protein was separated by SDS polyacrylamide gel electrophoresis and transferred onto polyvinylidene fluoride membrane. After the membrane was blocked, it was incubated at 4°C overnight with primary anti‐rabbit polyclonal antibodies purchased from Abcam Inc: STK4 (1:1000, ab51134), YAP (1:1000, ab226817), p‐YAP (1:1000, ab172374), LATS1 (1:5000, ab70561), Beclin1 (1:1000, ab217179), LC3B (1:3000ab51520), and Caspase‐3 (1:500, ab13847). After that, the membrane was further incubated with horseradish peroxidase‐labelled goat anti‐rabbit IgG secondary antibody (1:1000, Wuhan Boster Biological Technology Co., Ltd.) at 37°C for 1 hour and washed with PBS 3 times (5 minutes each time) for the colour development. Relative expression of target protein = grey value of target protein band/grey value of internal reference band in the same sample (GAPDH served as the internal reference).

### 2.12 Monodansylcadaverine (MDC) staining

After cell grouping and treatment, the cells were inoculated into a 6‐well plate containing coverslips before staining. After the treatment, the culture medium was discarded. The cells were gently washed 3 times with 1 × PBS buffer for 5 minutes each time, then 1 mL 1 × PBS buffer, and 1 μL prepared 100 mmol/L MDC storage solution were added to each well. About 100 μmol/L MDC working solution was prepared. The 6‐well plate was wrapped with tin foil and incubated in an incubator for 30 minutes without exposure to light. Finally, the PBS solution containing MDC was aspirated, and the cells were washed with 1 × PBS buffer 2 times, each time for 5 minutes. The autophagosome was observed under a fluorescence microscope.

### 2.13 **Terminal deoxynucleotidyl transferase (TdT)‐mediated 2**′**‐deoxyuridine 5**′**‐triphosphate (dUTP) nick end labelling (TUNEL)**

Samples fixed with neutral formaldehyde were dehydrated, embedded in paraffin and sliced. Samples were dewaxed twice by xylene (each time for 5 minutes), then dehydrated by 100%, 95%, 90%, 80%, 70% gradient ethanol, and washed 3 times with PBS (each time for 5 minutes). According to instructions of TUNEL detection kit (Roche, Basel, Switzerland), 50 μL TUNEL reaction solution (enzyme concentration solution and labelling solution at the ratio of 1:9) was added for 50‐min interaction. A total of 50 μL transforming agent‐peroxidase was added and incubated at 37°C for 30 minutes. Then, cells were incubated with 100 μL Diaminobenzidine solution for 10 minutes for the colour development. The cells were then counterstained with haematoxylin for 3 seconds and sealed with neutral gum. Five fields of vision were observed in each group. The apoptosis rate in each field of vision = (apoptotic cells/total number of cells) × 100%.

### 2.14 **5‐Ethynyl‐2**′**‐deoxyuridine (EdU) assay**

Cells in logarithmic growth phase were inoculated into a 96‐well plate at 4 × 10^3^ cells per well and cultured to normal growth stage. The EdU solution was diluted using cell culture medium at the ratio of 1000:1 to prepare a proper amount of 50 μm EdU culture medium. Each well was added with 100 μL 50 μM EdU culture medium and incubated for 2 hours. Then, the culture medium was abandoned. The cells were washed with PBS 1‐2 times (5 minutes each time). Each well was incubated for 30 minutes at room temperature with 50 μL cell fixative (PBS containing 4% paraformaldehyde), and then, the fixative was discarded. Each well was then added with 50 μL 2 mg/mL glycine and incubated in a decolorizing shaker for 5 minutes, and then, the glycine solution was removed. Each well was added with 100 μL penetrant (0.5% Tritonx‐100 PBS) and incubated for 10 minutes in a decolorizing shaker. The cells were washed with PBS once for 5 minutes. Each well was added with 100 μL 1 × Apollo^®^ staining solution. After the cells were incubated for 30 minutes at room temperature in a decolorizing shaker without exposure to light, the staining solution was then discarded. A total of 100 μL penetrant (0.5% Tritonx‐100 PBS) was added to allow decoloration in the decolorizing shaker 2‐3 times (10 minutes each time). Subsequently, the penetrant was discarded. Each well was added with 100 μL methanol for cleaning 1‐2 times with 5 minutes each time. The cells were washed with PBS once for 5 minutes. The reagent F was diluted with deionized water at a ratio of 100:1 to prepare 1 × Hoechst33342 reaction solution and stored in the dark. Each well was added into 100 μL 1 × Hoechst33342 reaction solution after incubation for 30 minutes at room temperature in a decolorizing shaker, and the reaction solution was discarded. Each well was added with 100 μL PBS for cleaning 1‐3 times.

### 2.15 Tumour xenografts in nude mice

Female BALB/C nude mice (4‐6 weeks old, no specific pathogen) were purchased from Sichuan University Medical Laboratory Animal Center). The cells were inoculated into a low‐adhesion culture plate for 7 d and centrifuged. After the supernatant was discarded, the cells were added with 1 mL 0.5% trypsin and cultured in a 37°C incubator. After that, the cell spheres were dissociated into single cells and the detachment was stopped by adding 3 mL of complete culture medium to collect cell precipitation. The normal saline was added, and the cells were made into single‐cell suspension. A total of 2 × 10^6^ cells were resuspended with 50 μL saline and then mixed with 50 μL Mat Rigel Matrix (1:1). The mixture was inoculated under the skin of nude mice. After inoculation, the tumour volume on the 0, 7th d, 14th, 21st d, 27th d and 35th d was observed and recorded. Tumour volume (mm^3^) = ½ × (L × W^2^), L represents the long diameter of the tumour, and W is the short diameter of the tumour.^19^ After 35 d of inoculation, nude mice were sacrificed, the tumours were peeled off and weighed, and then the tumours of each group were compared and photographed.

### 2.16 Statistical analysis

Data were analysed using SPSS 21.0 (IBM Corp) statistical software. The measurement data were expressed by mean ± standard deviation. The *t* test was used for data analysis between two groups. One‐way analysis of variance (ANOVA) was used for comparisons among multiple groups. And the comparison of data at different time points was analysed by repeated measures ANOVA. Kolmogorov‐Smirnov method was used to test the normality of data. One‐way ANOVA was used to compare the data with normal distribution among groups. Tukey's post hoc test was applied to post checking. The data of skewed distribution were analysed by non‐parametric test Kruskal‐Wallis, and post checked by Dunn's Multiple Comparison. A *P* value < .05 indicated that the difference was statistically significant.

## 3 RESULTS

### 3.1 LncRNA TNRC6C‐AS1 may affect STK4 expression and regulate the Hippo signalling pathway to regulate apoptosis and autophagy of TC

**Figure 1:**
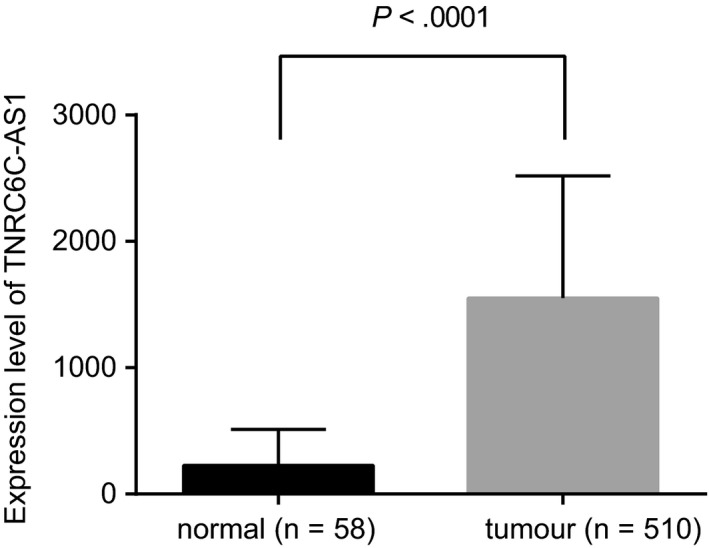
LncRNA TNRC6C‐AS1 is highly expressed in TC tissues. The expression of lncRNA TNRC6C‐AS1 in TC tissues (n = 510) and adjacent tissues (n = 58) analysed by TCGA database. The comparison between the two groups was analysed by *t* test, and data were expressed by mean ± standard deviation

Gene expression data of TC in TCGA database showed that the expression of lncRNA TNRC6C‐AS1 in TC was significantly higher than that in the normal control (Figure 1). The target gene and function of lncRNA TNRC6C‐AS1 were predicted through MEM and KEGG websites. Results showed that lncRNA TNRC6C‐AS1 might regulate the expression of STK4 and the Hippo signalling pathway to regulate apoptosis and autophagy of TC cells in vivo (Table 3).

**Table 3:** Target gene prediction and functional analysis of TNRC6C‐AS1

Pathway	*P* value	Gene
Phosphatidylinositol signalling system—Homo sapiens (human)	.000246	PRKCB; DGKD
Insulin resistance—Homo sapiens (human)	.003173	PRKCB; PPP1R3E
Phospholipase D signalling pathway—Homo sapiens (human)	.008345	DGKA; PTK2B
Other glycan degradation—Homo sapiens (human)	.009269	HEXDC; ENGASE
Hippo signalling pathway—Homo sapiens (human)	.011094	STK4
Choline metabolism in cancer—Homo sapiens (human)	.012023	DGKA; PRKCB
Non‐small cell lung cancer—Homo sapiens (human)	.013985	PRKCB; STK4

### 3.2 The silencing of lncRNA TNRC6C‐AS1 inhibits proliferation as well as promotes apoptosis and autophagy of TC cells

**Figure 2:**
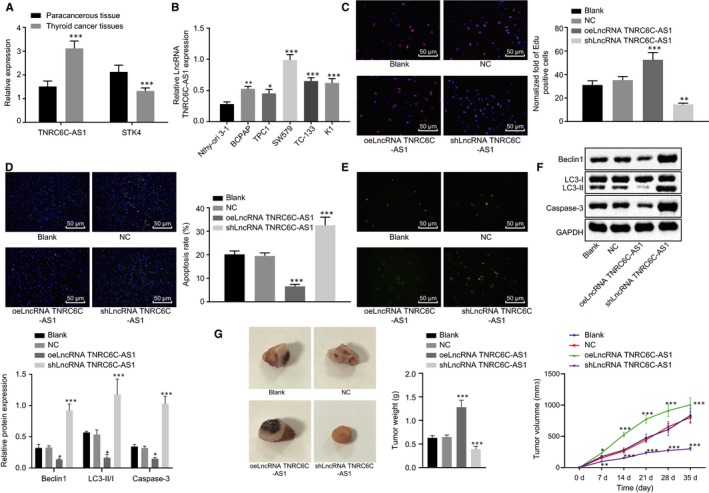
LncRNA TNRC6C‐AS1 silencing inhibits proliferation and promotes apoptosis and autophagy of TC cells in vivo. A, The expression of lncRNA TNRC6C‐AS1 and STK4 in 54 cases of TC tissues and adjacent tissues were analysed. ***P < .001 vs the adjacent tissues, (n = 54), the comparison between the two groups was analysed by *t* test (mean ± standard deviation). B, The expression of lncRNA TNRC6C‐AS1 in 5 TC cell lines was determined by RT‐qPCR, with normal human thyroid cell Nthy‐ori 3‐1 as a reference. *P < .05, **P < .01, ***P < .001 vs Nthy‐ori 3‐1. C, EdU assay was used to detect the proliferation ability of SW579 cells transfected with oelncRNA TNRC6C‐AS1 and shlncRNA TNRC6C‐AS1, respectively. D, TUNEL method was used to detect the apoptosis rate of SW579 cells transfected with oelncRNA TNRC6C‐AS1 and shlncRNA TNRC6C‐AS1, respectively. E, MDC method was used to detect the number of autophagic vesicles in SW579 cells transfected with oelncRNA TNRC6C‐AS1 and shlncRNA TNRC6C‐AS1, respectively, compared with the NC group. F, Western blot analysis was performed to assess the expression of apoptosis (Casepase‐3) and autophagy‐related (Beclin1 and LC3‐II/I) factors in SW579 cells transfected with oelncRNA TNRC6C‐AS1 and shlncRNA TNRC6C‐AS1, respectively, normalized to GAPDH. G, The representative pictures, weight and volume of tumours in nude mice (n = 14). *P < .05, **P < .01, ***P < .001 vs the NC group. The comparison among multiple groups was analysed by one‐way or repeated measures ANOVA (mean ± standard deviation). Values were obtained from 3 independent experiments in triplicate

RT‐qPCR of 54 TC tissues and adjacent tissues (Figure 2A) demonstrated that lncRNA TNRC6C‐AS1 expression in TC tissues was significantly higher, and STK4 expression was significantly lower in TC compared to those in adjacent tissues (*P* < .05). The expression of lncRNA TNRC6C‐AS1 (Figure 2B) in TC cell lines BCPAP, TPC1, SW579, FTC‐133, and K1 and normal thyroid cell line Nthy‐ori 3‐1, significantly increased in TC cell lines, and was highest in SW579 cells (*P* < .05) when compared with Nthy‐ori 3‐1 cells. EdU assay (Figure 2C), TUNEL (Figure 2D) and MDC staining (Figure 2E) results showed that there was no significant difference between the blank group and the NC group (*P* > .05). When compared with the NC group, shlncRNA TNRC6C‐AS1 was able to inhibit cell proliferation, promoted cell apoptosis and enhanced the green dot fluorescence brightness while oelncRNA TNRC6C‐AS1 was able to promote cell proliferation, inhibit cell apoptosis and suppress the green dot fluorescence brightness (*P* < .05). Western blot analysis was performed to determine expression of apoptosis (Casepase‐3) and autophagy‐related factors (Beclin1 and LC3‐II/I), followed by investigation on tumour formation in vivo. The results showed that delivery of oelncRNA TNRC6C‐AS1 induced significantly lower levels of Beclin1, LC3‐II/I and Casepase‐3 (Figure 2F) along with larger volume and weight (Figure 2G). Opposite changing tendency was observed following delivery of shlncRNA TNRC6C‐AS1. Therefore, lncRNA TNRC6C‐AS1 could promote proliferation and inhibit apoptosis and autophagy of TC cells.

### 3.3 LncRNA TNRC6C‐AS1 promotes STK4 methylation

**Figure 3:**
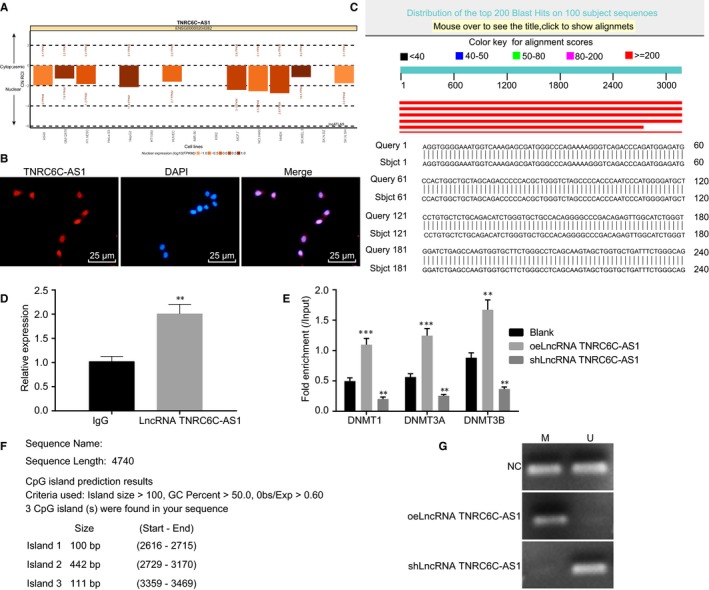
lncRNA TNRC6C‐AS1 promotes STK4 methylation A, lncATLAS website was applied to predict the subcellular localization of lncRNA TNRC6C‐AS1. B, FISH was used to detect lncRNA TNRC6C‐AS1 localization. C, Results of Blast comparison between lncRNA TNRC6C‐AS1 and STK4. D, ChIP was used to assess expression of lncRNA TNRC6C‐AS1 (**P < .01 vs the IgG group). E, RIP was used to detect the binding between lncRNA TNRC6C‐AS1 and methyltransferase. F, The distribution of CpG islands in promoter region of STK4 analysed by bioinformatics. G, MSP was used to detect methylation of STK4 (U: Unmethylation, Non‐methylation; M: Methylation). **P < .01, ***P < .001 vs the blank group. The comparison between two groups was analysed by *t* test while comparison among multiple groups should be statistically analysed by one‐way ANOVA (mean ± standard deviation). Values were obtained from 3 independent experiments in triplicate

The bioinformatics tool (http://lncatlas.crg.eu/) was employed to acquire the subcellular localization information. The analysis revealed that lncRNA TNRC6C‐AS1 was localized in the nucleus (Figure 3A). Based on FISH experiment results, lncRNA TNRC6C‐AS1 was further verified that its localization in the nucleus (Figure 3B). Blast comparison results showed that the promoter regions of lncRNA TNRC6C‐AS1 and STK4 had base complementary pairing binding sites (Figure 3C). In order to verify the binding of STK4 promoter region with methyltransferase, ChIP analysis of STK4 promoter region in SW579 cells was performed. The expression of lncRNA TNRC6C‐AS1 in the enriched product was identified and found that the expression of lncRNA TNRC6C‐AS1 was significantly higher than that of IgG NC (Figure 3D). The binding of lncRNA TNRC6C‐AS1 to methyltransferase was also analysed by RIP experiment. The results showed that when compared with the blank group, the combination with DNMT1, DNMT3a and DNMT3b was significantly increased in the oelncRNA TNRC6C‐AS1 group (*P* < .05), but decreased significantly in the shlncRNA TNRC6C‐AS1 group (*P* < .05) (Figure 3E). It was also found that the promoter region of STK4 existed variable methylation in the cytosine‐phosphate‐guanine (CpG) island by bioinformatics analysis (Figure 3F). MSP (Figure 3G) verified that the STK4 was highly methylated in SW579 cells. The above results showed that the expression of STK4 was regulated by lncRNA TNRC6C‐AS1, and lncRNA TNRC6C‐AS1 silenced STK4, hence allowing STK4 promoter methylation.

### 3.4 LncRNA TNRC6C‐AS1 silencing blocks the activation of Hippo signalling pathway via STK4

**Figure 4:**
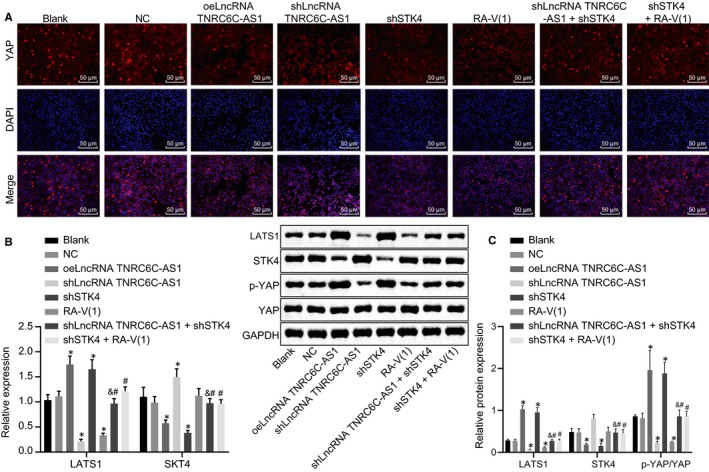
LncRNA TNRC6C‐AS1 silencing disrupts the activation of the Hippo signalling pathway via STK4 in SW579 cells. A, Immunofluorescence detection of YAP nucleus entering in SW579 cells after transfection. B, The expression of LATS1 and STK4 in SW579 cells normalized to GAPDH after transfection was determined by RT‐qPCR. C, Western blot analysis for the determination of STK4 and YAP protein levels and the extent of YAP phosphorylation in SW579 cells normalized to GAPDH after transfection. *P < .05 vs the blank group, #*P* < .05 vs the shSTK4 group, &*P* < .05 vs the shlncRNA TNRC6C‐AS1 group. The comparison among multiple groups was analysed by one‐way ANOVA (mean ± standard deviation). Values were obtained from 3 independent experiments in triplicate

The small molecule inhibitor RA‐V(1) of Hippo signalling pathway was used to further verify the role of Hippo signalling pathway in TC cells. The number of YAP entering the nucleus is an important indicator of Hippo signalling pathway activation. The nuclear translocation of YAP was detected by cell immunofluorescence staining, and it was found that in the TC cell line SW579, the number of YAP entering the nucleus decreased significantly in the shSTK4 and oelncRNA TNRC6C‐AS1 group; while in the oelncRNA TNRC6C‐AS1 or RA‐V(1) group with signalling pathway inhibitor, the number of YAP entering the nucleus increased significantly. Meanwhile, when lncRNA TNRC6C‐AS1 was silenced and STK4 was inhibited, nuclear translocation of YAP was reduced than STK4 inhibition alone and elevated than lncRNA TNRC6C‐AS1 silence alone. When compared with STK4 inhibition alone, the additional inactivation of the Hippo signalling pathway decreased YAP importation into nucleus (Figure 4A) (*P* < .05).

At the same time, the mRNA expression of LATS1 and STK4 was determined using RT‐qPCR and the protein levels of STK4, LATS1 and YAP, as well as the extent of YAP phosphorylation was determined by Western blot analysis. The results showed that the mRNA and protein levels of STK4 decreased significantly, and the mRNA and protein levels of LATS1 increased while the extent of YAP phosphorylation increased significantly, and Hippo signalling pathway was activated in the shSTK4 and oelncRNA TNRC6C‐AS1 groups. However, it was reciprocal in the shlncRNA TNRC6C‐AS1 group (Figure 4B‐D) (*P* < .05). The addition of RA‐V(1) decreased mRNA and protein levels of LATS1 and the extent of YAP phosphorylation. In comparison with shSTK4 treatment alone, the co‐transfection of shlncRNA TNRC6C‐AS1+shSTK4 and shSTK4+RA‐V(1) also induced lower mRNA and protein levels of LATS1 and reduced extent of YAP phosphorylation. However, shlncRNA TNRC6C‐AS1 increased the mRNA and protein levels of LATS1 and the extent of YAP phosphorylation with shSTK4. Taken together, lncRNA TNRC6C‐AS1 could mediate the activation of the Hippo signalling pathway via STK4.

### 3.5 LncRNA TNRC6C‐AS1 silencing promotes apoptosis and autophagy of TC cells through STK4/Hippo axis

**Figure 5:**
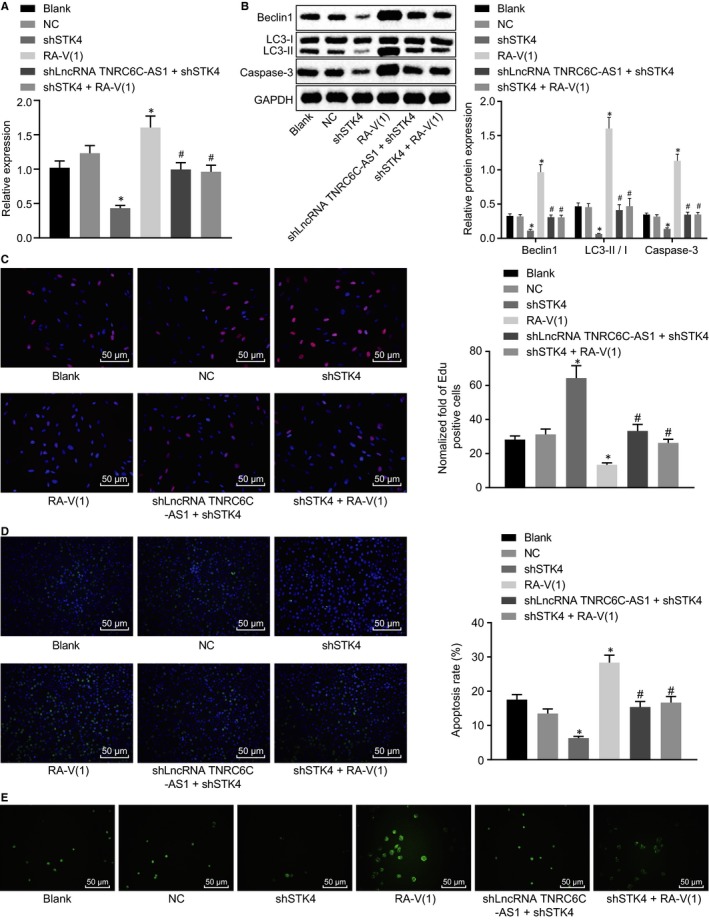
LncRNA TNRC6C‐AS1 silencing promotes the apoptosis and autophagy of TC cells through STK4/Hippo. A, The mRNA expression of Beclin1 in transfected SW579 cells normalized to GAPDH was determined by RT‐qPCR. B, Western blot analysis was used to determine the protein levels of Beclin1, LC3‐II/I and Casepase‐3 in SW579 cells normalized to GAPDH after transfection. C, The proliferation ability of SW579 cells in each group was tested by EdU assay. D, TUNEL method was used to detect the apoptosis rate of SW579 cells. E, MDC method was used to detect the number of autophagic vesicles of SW579 cells in each group. *P < .05 vs the blank group, #*P* < .05 vs the shSTK4 group. The comparison among multiple groups was analysed by one‐way ANOVA (mean ± standard deviation). Values were obtained from 3 independent experiments in triplicate

RT‐qPCR and Western blot analysis were carried out to determine effect of lncRNA TNRC6C‐AS1 on the apoptosis and autophagy of TC cells through STK4/Hippo axis. When compared with the blank group and the NC group, mRNA and protein levels of Beclin1 decreased, the protein levels of LC3‐II/I and Casepase‐3 were reduced significantly (Figure 5A‐C), the number of autophagic vesicles decreased significantly and the proliferation ability was enhanced obviously, the ability of apoptosis and autophagy was declined significantly in the shSTK4 group (Figure 5D‐E) (all *P* < .05). However, in the RA‐V(1) group, after blocking Hippo signalling pathway, mRNA and protein levels of Beclin1 increased significantly and the protein levels of LC3‐II/I and Casepase‐3 increased significantly, the number of autophagic vesicles increased significantly, the ability of proliferation decreased remarkably and the ability of apoptosis and autophagy increased significantly (all *P* < .05). Among them, Beclin1 and LC3‐II/I were related to autophagy, high levels indicating high autophagy ability (*P* < .05). Nevertheless, shSTK4 suppressed cell apoptosis and autophagy with shlncRNA TNRC6C‐AS1 or RA‐V(1) more obviously (*P* < .05). To conclude, lncRNA TNRC6C‐AS1 could mediate TC cell apoptosis and autophagy via the STK4/Hippo axis.

### 3.6 LncRNA TNRC6C‐AS1 silencing suppresses tumour formation and the growth of TC cells in vivo through STK4/Hippo axis

**Figure 6:**
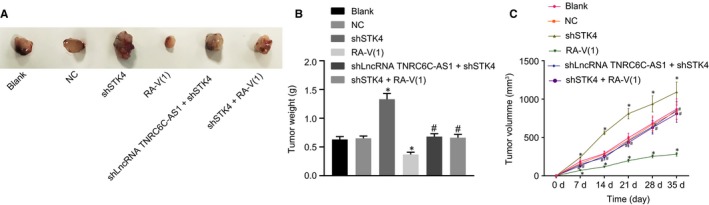
LncRNA DLX6‐AS1 silencing inhibits tumorigenesis and tumour growth of TC cells in vivo through STK4/Hippo. A, Representative pictures of tumours of nude mice with different cells after transfection. B, Tumour weight in nude mice. C, Tumour volume in nude mice. *P < .05 vs the blank group, # *P* < .05 vs the shSTK4 group. The comparison among multiple groups was analysed by one‐way ANOVA. The comparison of data at different time points was analysed by repeated measures ANOVA (mean ± standard deviation). Values were obtained from 3 independent experiments in triplicate

The results of mouse subcutaneous tumour‐bearing model were established to validate the effect of silencing lncRNA TNRC6C‐AS1 on tumour formation and growth of TC cells in vivo. From the 7th d, when compared with the blank group and the NC group, the shSTK4 group had earlier tumour formation, faster growth rate and larger tumour volume. While in the RA‐V(1) group, the tumour formation and the growth rate were slower, and the tumour volume was smaller (*P* < .05). Nevertheless, compared with shSTK4 treatment alone, the presence of shlncRNA TNRC6C‐AS1 or RA‐V(1) with shSTK4 significantly slowed down tumour growth (*P* < .05) (Figure 6A‐C). These findings validated the suppressive role of silencing lncRNA TNRC6C‐AS1 in vivo.

## 4 DISCUSSION

TC is the most common endocrine malignancy, and the incidence has increased in both developed and developing countries.^20^ TC is a major health threat to human beings all over the world.^21^ Meanwhile, a new study has showed the influence of lncRNA TNRC6C‐AS1 on proliferation, apoptosis and invasion of papillary TC cells.^8^ In this study, we explored the role of the lncRNA TNRC6C‐AS1, STK4 and Hippo signalling pathway in TC cells. Consequently, this study demonstrated that lncRNA TNRC6C‐AS1 silencing in TC cells inhibited the methylation of STK4 promoter, up‐regulated STK4 expression and suppressed Hippo signalling pathway, thus inhibiting the proliferation of TC cells and promoting cell apoptosis and autophagy in TC.

In this study, we found that lncRNA TNRC6C‐AS1 expression in TC tissues was significantly increased and STK4 expression was significantly decreased. A study has approached that lncRNA TNRC6C‐AS1 is up‐regulated in TC tissues and cells,^9^ which is consistent with our results. STK4 is the key kinase participated in the Hippo signalling pathway.^22^ A study has showed that the protein level of STK4 decreases in the progression of prostate cancer.^23^ Based on RIP assay in this study, the expression of STK4 was regulated by lncRNA TNRC6C‐AS1. LncRNA TNRC6C‐AS1 promoted STK4 by inhibiting the methylation of STK4 promoter, thus inhibiting the phosphorylation level of YAP and Hippo signalling pathway. Hippo signalling pathway is a development control signal pathway, which plays a regulatory role in cell proliferation and apoptosis.^24^ YAP is considered as the main effector of the Hippo signalling pathway in various organisms and tissues.^25^ Knockdown of lncRNA GHET1 suppresses the cell proliferation and invasion by down‐regulating YAP1 expression in the non‐small cell lung cancer.^26^ YAP‐1 functions as an oncogenic and a putative prognostic biomarker of recurrence in patients with PTC.^27^

Furthermore, in the RA‐V(1) group, after the blocking of Hippo signalling pathway, the expression of LATS1 and the extent of YAP phosphorylation decreased, the expression of Beclin 1 increased, expression of LC3‐II/I and Casepase‐3 increased, and the number of autophagic vesicles increased, thus inhibiting proliferative capacity and promoting apoptotic and autophagic ability. LATS1 is an upstream protein of YAP and adjusts YAP by removing it from the nuclear chamber.^28^ A study has showed that LATS proteins are notably down‐expressed in breast cancer and non‐small cell lung cancer.^29^ Dephosphorylated YAP allows its entry into nucleus where it activates the transcription of pro‐proliferative and anti‐apoptotic targets.^25^ YAP can be phosphorylated and made inactive by alive LATS1.^30^ A study has showed that down‐regulated lncRNA taurine‐up‐regulated gene 1 (TUG1) occurs with decreased YAP levels.^31^ A new study has noted that Beclin1 works in autophagy via complexes like the Beclin1‐PI3KC3 or Beclin1‐Bcl‐2 complex.^32^ LC3II was considered as an autophagy‐linked marker in cell tissues.^33^ YAP plays a role in the processes of autophagy and apoptosis 34 and silencing YAP increased the ratio of LC3‐I and LC3‐II, accompanied by the autophagy‐related protein Beclin1.^15^ Caspase‐3 plays an important role in the caspase cascade reaction and represents a major enzyme and promoter of apoptosis in cancer cells.^35^ A new study has determined a point that multiple myeloma cell apoptosis is accelerated by the improvement of Caspase‐3 activity.^36^

**Figure 7:**
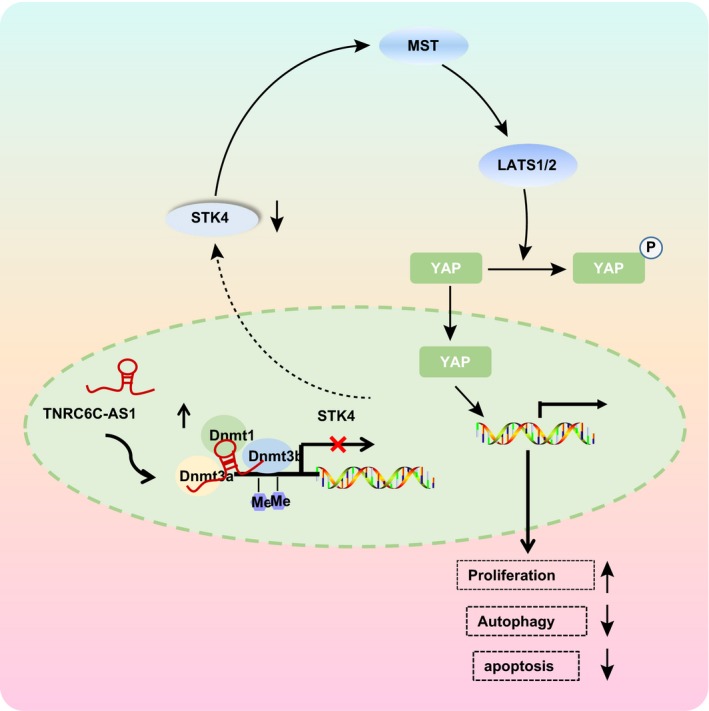
LncRNA TNRC6C‐AS1 silencing inhibits proliferation and promotes apoptosis and autophagy of TC cells. LncRNA TNRC6C‐AS1 is up‐regulated in TC cells, by binding DNMT to STK4 promoter region, which further promotes the methylation of STK4 promoter, thereby down‐regulating STK4 and activating Hippo signalling pathway. This further promotes the proliferation of TC cells and inhibits the apoptosis and autophagy of TC cells

This study has supported that lncRNA TNRC6C‐AS1 silencing inhibits STK4 promoter methylation, up‐regulates the expression of STK4 and inactivates the Hippo signalling pathway, thus suppressing the proliferation and promoting cell apoptosis and autophagy of TC cells (Figure 7). However, due to the limited simple size and experimental conditions, clinical analysis should be conducted in the future in order to validate the results of this study and excavate the predictive values of lncRNA TNRC6C‐AS1 in diagnosis and prognosis of TC. We speculate that lncRNA TNRC6C‐AS1 or STK4 is a promising therapeutic target for the treatment of TC. Therefore, the effects of STK4 methylation on TC need to be further verified as it is far too little researched.

## CONFLICT OF INTEREST

We declare that we have no conflicts of interest.

## AUTHOR CONTRIBUTIONS

Xinzhi Peng, Chengcheng Ji and Langping Tan designed the study. Shaojian Lin collated the data, Yue Zhu designed and developed the database, Miaoyun Long carried out data analyses and produced the initial draft of the manuscript. Dingyuan Luo, Honghao Li and Xinzhi Peng contributed to drafting the manuscript. All authors have read and approved the final submitted manuscript.

## Data Availability

The data that support the findings of this study are available from the corresponding author upon reasonable request.
